# Phenolic Composition, Antioxidant and Anticancer Potentials of Extracts from *Rosa banksiae* Ait. Flowers

**DOI:** 10.3390/molecules25133068

**Published:** 2020-07-06

**Authors:** Chen Zeng, Siyuan Luo, Shiling Feng, Tao Chen, Lijun Zhou, Ming Yuan, Yan Huang, Jinqiu Liao, Chunbang Ding

**Affiliations:** College of Life Science, Sichuan Agricultural University, Ya’an 625014, China; zc15183819422@126.com (C.Z.); luosiyuan1998@163.com (S.L.); fengshilin@outlook.com (S.F.); chentao293@163.com (T.C.); zhouzhou124@126.com (L.Z.); yuanming@sicau.edu.cn (M.Y.); shirley11hy@163.com (Y.H.); liaojinqiu630@sicau.edu.cn (J.L.)

**Keywords:** *Rosa banksiae* Ait., fractionation extraction, polyphenols, HPLC

## Abstract

*Rosa banksiae* Ait. (*R. banksiae*) is a traditional Chinese folk medicine and an ornamental plant. Most previous studies have focused on cultivation and utilization while there are few research papers on the pharmacological activity of *R. banksiae*. This study aimed to get a better understanding of *R. banksiae* by extracting polyphenols with fractionated extraction technology. The results showed that ethyl acetate phase (EAP) contained the most polyphenols, while water phase (WP) had the least. HPLC analysis indicated that rutin and luteolin-4′-*O*-glucoside existed in the EAP and butanol phase (BP), but quercetin was only detected in the EAP. Six phenolic compositions were not detected in WB. The antioxidant and anti-tumor abilities of the EAP and BP were excellent. The results revealed that *R. banksiae* possessed a great antioxidant capacity and was rich in polyphenols, thus indicating *R. banksiae* was suitable for being a natural antioxidant and an abundant source of polyphenols.

## 1. Introduction

In the process of human metabolism, a large number of free radicals will be produced. They have strong oxidation, which will cause damage to the function of biofilms and cells in the human body, thereby increasing the risk of cardiovascular disease and cancer, and accelerating the process of human aging [1]. Polyphenols, such as EGCG, quercetin, luteolin and others, were proved to be the principal antioxidants [2,3,4]. Polyphenols are a kind of secondary metabolites with polyphenol structure which exist widely in plants, and have highly efficient bioactivities. They possess strong antioxidant abilities [5], with obvious effects of anti-mutation [6], anti-cancer [7], and inhibiting hypertensive [8]. Sasaki et al. found that polyphenols were able to protect cells from this kind of mutations, as they can absorb ultraviolet light from sunlight [9]. Polyphenols from betel nut tannins have been used to fight tooth decay, because they bind to the proteins of microbes and viruses, leading to the deactivation of proteins [10]. These bioactivities have something to do with inhibition of microbial metabolic enzymes or complexation of trace metal ions by polyphenols.

*Rosa banksiae* Ait. (*R. banksiae*), belonging to Rose family, is a rosaceous perennial evergreen vine, growing to about 20 feet tall, with a long lifespan. *R. bankisae* is an important, widespread woody plant and a special species in southwest China. As early as 1708, it was recorded in *Guang Qunfang Pu* an ancient book recording flowers written by Wang Hao in Qing dynasty. In traditional Chinese medicine, this plant is effective at relieving pain and stopping bleeding. However, there are few literary reports on pharmacological activity about *R. banksiae*. At present, most of the research on *R. banksiae* are focusing on cultivation and utilization, physiology biochemistry and genetic diversity [11,12]. Yu et al. found the chemical compositions of the essential oils from *R. banksiae* flowers were made up with alcohol, terpenoid, ketones and lipid [13]. Some flavonol aglycones were found in the leaves [14]. However, it is worth mentioning that the chemical composition and relative bioactivities of related extracts from this plant have not yet been researched.

In our prophase research, we have found that the flowers from *R. banksiae* are used as a raw material for making pastry, such as moon cakes, and extracts of *R. banksiae* contained a certain level of polyphenols. Surprisingly, related research results have been seldom reported. Thus, the goal of this present study was to identify phenolic compounds of *R. banksiae* and test their antioxidant activity, hoping this research may help us to gain a better understanding of phenolic compounds from *R. banksiae* for industrial applications and pharmacological activities analysis.

## 2. Results and Discussion

### 2.1. Polyphenols Content in Different Extraction Phases

The content of polyphenols from *R. banksiae* in different extraction phases were shown in Table 1. The content in ethyl acetate phase (EAP) was the highest (759.69 ± 21.54 mg/g), about 15 times higher than that in water phase (WP) (48.99 ± 1.29 mg/g). The content of polyphenols in butanol phase (BP) was 660.75 ± 22.05 mg/g. The polyphenols contents in EAP and BP were significantly higher than that in WP with *p* value < 0.001. In addition, EAP contained more than BP with *p* value < 0.01. Methanol and ethanol have often been used as a solvent to extract total polyphenol from plants and the yield was ranging from 2.93 mg/g to 160.55 mg/g [4]. In this work, the fractional extraction method could highly enrich the content of polyphenols in butanol and ethyl acetate phase, respectively. The polarity of ethyl acetate phase was larger than the butanol phase. Hence, the result indicated that the polarity of polyphenols in *R. banksiae* tended to be large and was more similar to the polarity of ethyl acetate. 

### 2.2. Analysis of HPLC

**Figure 1:**
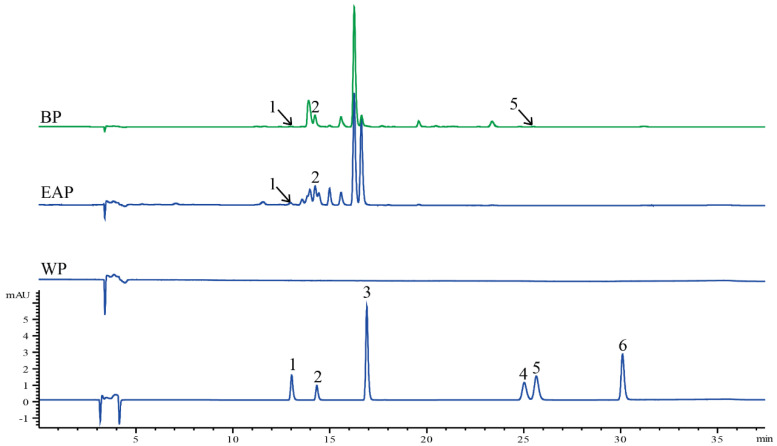
The high performance liquid chromatography (HPLC) of mixed standard. Rutin (1), luteolin-4′-*O*-glucoside (2), apigenin-7-O-glucoside (3), luteolin (4), quercetin (5), and apigenin (6).

The HPLC chromatogram of standards was given in Figure 1, and the mixed standards were rutin (1), luteolin-4′-*O*-glucoside (2), apigenin-7-O-glucoside (3), luteolin (4), quercetin (5), and apigenin (6). Three compositions were identified in three phases by matching their retention times against those of the standards (Figure 1), such as rutin (1), luteolin-4′-*O*-glucoside (2) and quercetin (5). No composition in the mixed standard was detected in WP (Figure 1).

The phenolic compositions of three phases were shown in Table 2. Rutin only existed in ethyl acetate phase (10.99 ± 1.92 mg/g) and butanol phase (11.06 ± 2.20 mg/g). The rutin content in ethyl acetate and butanol phases was not significantly different according to student’s T test (*p* > 0.05). The luteolin-4′-*O*-glucoside in ethyl acetate phase was significantly higher than butanol phase with *p* value < 0.001. However, the quercetin was only detected in ethyl acetate phase (5.73 ± 0.24 mg/g).

Polyphenols wildly exist in rosaceous plants. JE Lee et al. found 12 identified polyphenols compositions in chokeberry, such as quercetin 3-O-rutinoside, apigenin 7, 4′-di-*O*-rhamnoside and quercetin rhamnosylhexoside [15]. In this study, luteolin-4′-*O*-glucoside was found in *R. banksiae* with a high content. Therefore, *R. banksiae* could be a potential source for luteolin-4′-*O*-glucoside.

### 2.3. Analysis of Antioxidant Activity in Different Extraction Phases

**Figure 2:**
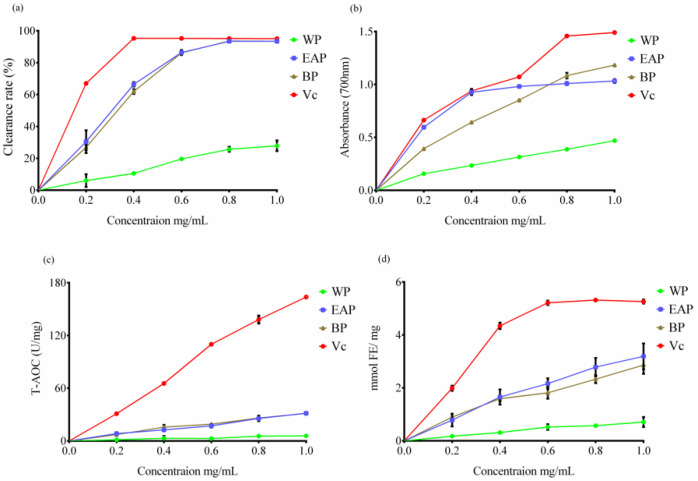
The antioxidant ability of three phases (**a**) 2,2-Diphenyl-1-picryl-hydrazyl (DPPH) free radical scavenging capacity, (**b**) total reduction capacity, (**c**) total antioxidant capacity (T-AOC) and (**d**) ferric ion reducing antioxidant power (FRAP).

DPPH free radical scavenging capacity was widely applied to assess the bioactivity of natural substances [16]. As shown in Figure 2a, the DPPH radical scavenging ability rates of three phases promoted with the increasing concentration and tended to be flatten at a high concentration. The clearance rate of water phase was lower than 30%, showing a weak antioxidant ability. The clearance rates of ethyl acetate phase and butanol phase were almost the same and close to Vc. The maximum clearance rates of three phases at 1.0 mg/mL were 27.85% (WP), 93.45% (EAP) and 93.46% (BP), respectively. IC_50_ was 1.43 mg/mL (WP), 0.31 mg/mL (EAP) and 0.34 mg/mL (BP), respectively.

An ascending absorbance meant a promoting total reducing power. Different extraction phases had certain total reduction capacity, showing a good dose-effect relationship with the increasing of sample concentration. As shown in Figure 2b, the absorbance of EAP and BP was higher than WP but lower than Vc. Three phases all had certain total antioxidant capacity, but much lower than positive control Vc (Figure 2c). When the concentration reached 1.0 mg/mL, the total antioxidant capacity of three phases were 5.905 U/mg (WP), 31.62 U/mg (EAP) and 31.42 U/mg (BP), respectively. Ferric ion reducing antioxidant power (FRAP) was widely used to screen high-antioxidant ability substances. The FRAP of EAP was not significantly different from BP, but both of them were significantly higher than WP (*p* < 0.0001). When the concentration reached 1.0 mg/g, the FRAP of three phases was 0.7114 mmol FE/mg (WP), 3.195 mmol FE/mg (EAP) and 2.862 mmol FE/mg (BP), respectively.

Polyphenols from natural plants have been proved to possess an effective antioxidant ability [16]. As shown in Table 1 and Table 2, EAP and BP contained a high content of polyphenols while WB had the lowest content. The antioxidant ability results showed the EAP and BP were higher than WP. Therefore, the antioxidant ability of *R. banksiae* may connect with polyphenols content. Lu et al. and Higdon et al. obtained similar results that indicate that polyphenols possess effective antioxidant ability [5,17]. Süzgeç-Selçuk et al. proved that the luteolin-4′-*O*-glucoside possessed an excellent antioxidant ability [18]. EAP and BP were rich in luteolin-4′-*O*-glucoside; hence, the great antioxidant activity of EAP and BP may relate to their high content of luteolin-4′-*O*-glucoside.

### 2.4. Analysis of Anti-Tumor Activity in Different Extraction Phases

**Figure 3:**
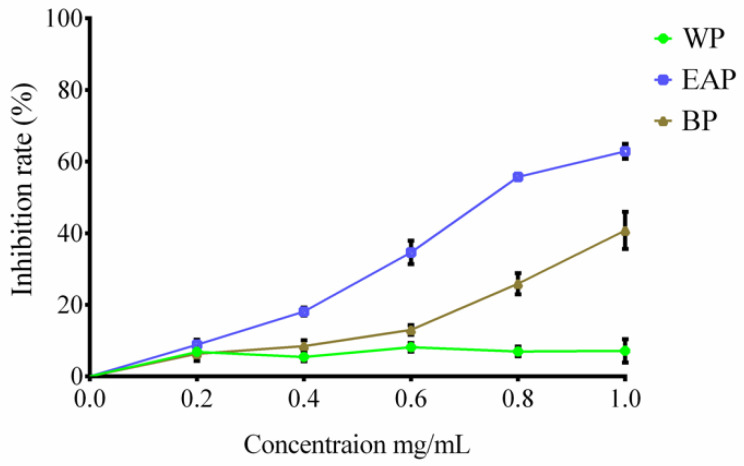
The inhibition effect of three phases on Hela cells treated for 48 h.

As shown in Figure 3, the growth of Hela cells treated with EAP and BP was obviously inhibited as the increasing sample concentrations. Nevertheless, WP exhibited a weak suppression ability on the proliferation of Hela cells. Particularly, EAP possessed a strong restrain effect compared with the other two phases. Polyphenols were proved to be the principal drugs against cancer [19]. As shown in Table 1, three phases contained different content of polyphenols. The content in WP was the lowest; therefore, WP could not restrain Hela cells. Luteolin-4′-*O*-glucoside was also reported to possess a strong inhibitory efficacy on Hela cells [20]. Moreover, EAP contained more luteolin-4′-*O*-glucoside than BP (Table 1); hence, the restrain effect of EAP was the strongest.

## 3. Materials and Methods

### 3.1. Materials and Reagents

Flowers from *Rosa banksiae* Ait. were picked up at the campus of Sichuan Agricultural University, Sichuan, Ya’an, China, at 29°58′49″ N, 120°59′19″ E.

2,2-Diphenyl-1-picryl-hydrazyl (DPPH) was purchased from Sigma Chemical Co. (St. Louis, MO, USA). Butanol, ethyl acetate and gallic acid were purchased from the Chengdu Kelong Chemical Factory (Chengdu, China). FRAP assay kit was purchased from Beyotime Biotechnology (Shanghai, China). Total antioxidant capacity assay kit was purchased from Nanjing Jiancheng Bioengineering Institute (Nanjing, China). All chemicals were analytical grade.

### 3.2. Fractionation Extraction of R. Banksiae Flowers

A quantity of 80 g *R. banksiae* flowers were mixed with 1 L water and extracted at 99 °C for 4 h. The water extract was filtered through a decompress filter, decanted in the separation funnel. Equal volume ethyl acetate was added for the first-time extraction for 2 h. After separating the liquid, the ethyl acetate phase of *R. banksiae* was obtained. Then, equal volume butanol was added to the water phase for the second-time extraction for 2 h. Then the separating liquid butanol phase was complete. Water phase (WP), ethyl acetate phase (EAP), and butanol phase (BP) were concentrated by a freeze drier. The concentrations were set at −20 °C for following experiments.

### 3.3. Determination of Polyphenols Content

The polyphenols content was measured by Folin-phenol reagent method. Briefly, 0.2 mL sample solution was mixed with 0.2 mL 25% folin-phenol reagent placed at dark for 5 min, then 0.2 mL 7.5% sodium carbonate (*w/v*) was added into the solution placed at dark for 5 min. Finally, the solution was diluted with up-water to 2 mL. After standing for 20 min, the absorbance of reaction solution was read at 700 nm. The polyphenols content was calculated according to the standard curve made with gallic acid as reference. The polyphenols content was expressed as milligram gallic acid equivalent per gram (mg GAE/g) of dry sample.

### 3.4. HPLC Analysis

Phenolic compositions in three phases were analyzed by high performance liquid chromatography (HPLC) with C18 column and HPLC system. The chromatographic analysis was performed using an Agilent 1260 HPLC (Agilent Technologies, Santa Clara, CA, USA) coupled with a UV-Vis DAD detector at 350 nm and a C18 reversed-phase column (5.0 µm, 150 mm × 4.6 mm). The phenolic compositions were analyzed by a gradient elution program using (A) Aqueous phosphoric acid (0.15%, *v/v*) and (B) acetonitrile (containing 0.1% acetic acid *v/v*) were used as the mobile phase and the gradient program was set as follows: 10–16% B (0–3 min), 16–30% B (3–20 min), 30–40% B (20–25 min), 40–16% B (25–30 min), and 16–10% B (30–40 min). The flow rate was 0.5 mL/min, and the temperature of the column was kept at 30 °C. The phenolic concentrations of rutin, luteolin-4′-*O*-glucoside, apigenin-7-O-glucoside, luteolin, quercetin, and apigenin were determined using a standard curve, and all results were presented as milligrams per gram (mg/g) of dry matter.

### 3.5. Determination of Antioxidant Activities

#### 3.5.1. Determination of DPPH Free Radical Scavenging Capacity

The three phase extracts of *R. banksiae* were resolved in water, ethyl acetate and butanol to different concentrations, respectively. The determination of DPPH radical scavenging ability of three extracts was based on the method described by Villano with slight modifications [14]. Briefly, 40 μL ascorbic acid (Vc) (0.2–1.0 mg/mL), which served as positive control, and *R. banksiae* samples (0.2–1.0 mg/mL) were respectively added into 140 μL DPPH ethanol solution (0.4 mM). The mixtures were incubated at room temperature in the dark for 10 min, then absorbance of mixture was measured at 517 nm by microplate reader (Spectramax M2, San Francisco, USA). The following formula was used to calculate the DPPH radical scavenging ability:

DPPH radical scavenging activity (%) = (1 − A1/A0) × 100%

where A1 is the absorbance of the reaction solution with the sample and A0 is the control group replaced with distilled water.

#### 3.5.2. Determination of Total Reduction Capacity

The total reduction capacity of *R. banksiae* was determined by the potassium ferricyanide reduction method. 0.2 mL of *R. banksiae* samples (0.2–1.0 mg/mL) were mixed with 0.5mL PBS (0.2 M) and 0.5 mL K_3_[Fe(CN)_6_] (1%, *w/v*). The mixtures were placed at 50 °C for 20 min, after that, 0.5 mL TCA (10%, *w/v*) was instantly added into and solutions were cooled at 0 °C for 5 min. A volume of 0.5 mL supernatant was mixed with 0.1 mL FeCl_3_ (0.1%, *w/v*) and 0.5 mL distilled water. After 10 min of standing at room temperature, the absorbance of reaction solution was read at 700 nm. The increasing of absorbance was meaning a promoting reducing power.

#### 3.5.3. Determination of Ferric Ion Reducing Antioxidant Power (FRAP) and Total Antioxidant Capacity (T-AOC)

The FRAP and T-AOC were tested by the FRAP and T-AOC assay kits, and the process was operated according to the kit instructions. Vc was used as the positive control. The results of FRAP were expressed as millimoles of ferrous sulfate equivalent per mg (mM FE/mg) of dry sample. The T-AOC results were expressed as unit per milligram (U/mg) of dry sample.

### 3.6. Anti-Cancer Assay

#### 3.6.1. Cell Culture

Hela cells were kindly provided by Stem Cell Bank, Chinese Academy of Sciences, Shanghai, China. Hela cells were cultured in dulbecco’s modified eagle medium (DMEM) containing 10% FBS (purchased from Gibco, Carlsbad, CA, USA) at 37 °C in a humidified atmosphere of 95% air and 5% CO_2_.

#### 3.6.2. Inhibition Effect on Hela Cells

The inhibition effect of three phases on the proliferation of Hela cells was determined by CCK-8 assay (purchased from Boster Biological Technology Co. Ltd., Wuhan, China). Briefly, three phases were resolved in DMSO, and filtered by 0.22 μm filter membrane. The cells were planted in 96-well microplate at the density of 10^5^ per milliliter and maintained at 37 °C for 6 h before added with sample solutions (the concentration of DMSO was less than 1%). After 48 h treatment, the solutions were completely replaced with new fresh medium. 10 μL CCK-8 was added into the wells and the plate was placed at 37 °C for 30 min before reading the absorbance at 450 nm. The inhibition rate was calculated according to the following formula:

Inhibition rate (%) = (1 − A1/A2) × 100%

where the A1 was the absorbance of treated group; A2 was the absorbance of control group.

### 3.7. Statistical Analysis

The data were analyzed by one-way analysis of variance (ANOVA) and Student’s test with GraphPad Prism 6 (GraphPad Software, Inc., La Jolla, CA, USA). All values were expressed as mean ± SD. All experiments were carried out in triplicate. *p* value less than 0.05 was considered as statistically significant.

## 4. Conclusions

Previous studies ignored the chemical compositions and pharmacological capacity of *Rosa banksiae*. However, the polyphenols content from *R. banksiae* flowers was firstly determined and shown to exist at a high value in EAP, followed by BP and WP. HPLC was successfully applied to identified phenolic components such as rutin, luteolin-4′-*O*-glucoside and quercetin in three phases. Furthermore, the antioxidant activities of EAP as well as BP were higher than WP and close to Vc. Moreover, EAP and BP showed a strong inhibitory efficacy on Hela cells compared with the effect of WP. This may be caused by the different polyphenols content in three phases. Above all, *Rosa banksiae* contained a high level of polyphenols and possessed a strong antioxidant ability, thus providing a theoretical basis for potentially economical utilization of *R. banksiae* in the field of food and health products. 

## Figures and Tables

**Table 1:** The polyphenols content of three phases.

Components	Polyphenol Content (mg GAE/g)
Water phase	48.99 ± 1.29
Ethyl acetate phase	759.69 ± 21.54 ***^##^
Butanol phase	660.75 ± 22.05 ***

Note: The polyphenols content was expressed as milligram gallic acid equivalent per gram (mg GAE/g) of dry sample. “***” means the polyphenols contents in ethyl acetate phase and butanol phase were significantly different from that in water phase with *p* value < 0.001. “^##^” means the polyphenols contents in ethyl acetate phase was significantly different from that in butanol phase with *p* value < 0.01.

**Table 2:** The phenolic compositions of three phases.

Phenolic Compositions	Regression Curve	Correlation Coefficient	Water Phase	Ethyl Acetate Phase	Butanol Phase
rutin	y = 2533.5x + 2.4153	0.999	nd	10.99 ± 1.92	11.06 ± 2.20 ^ns^
luteolin-4′-*O*-glucoside	y = 2240.2x + 0.8639	0.999	nd	368.83 ± 40.43	102.60 ± 4.04 ***
apigenin-7-*O*-glucoside	y = 5886.8x + 3.0264	0.998	nd	nd	nd
luteolin	y = 1978.4x + 1.8608	0.998	nd	nd	nd
quercetin	y = 4691.3x − 3.6269	0.999	nd	5.73 ± 0.24	nd
apigenin	y = 9596.4x + 1.5547	0.999	nd	nd	nd

Note: All results were expressed as milligram per gram (mg/g) of dry sample. “nd” means not detected. “ns” means the content of rutin in ethyl acetate and butanol phases is not significantly different by student’ T test. “***” means the luteolin-4′-*O*-glucoside in butanol phase is significantly different from that in ethyl acetate phase with *p* value < 0.001.

## References

[1] Cadenas E., Davies K.J.A. (2000). Mitochondrial free radical generation, oxidative stress, and aging. Free Radic. Biol. Med..

[2] Hirano R., Sasamoto W., Matsumoto A., Itakura H., Igarashi O., Kondo K. (2001). Antioxidant Ability of Various Flavonoids against DPPH Radicals and LDL Oxidation. J. Nutr. Sci. Vitaminol..

[3] Seelinger G., Merfort I., Schempp C.M. (2008). Anti-Oxidant, Anti-Inflammatory and Anti-Allergic Activities of Luteolin. Planta Med..

[4] Assefa A.D., Keum Y.S., Saini R.K. (2018). A comprehensive study of polyphenols contents and antioxidant potential of 39 widely used spices and food condiments. J. Food Meas. Charact..

[5] Lu Y., Yeap L.F. (2000). Antioxidant and radical scavenging activities of polyphenols from apple pomace. Food Chem..

[6] Katiyar S.K., Agarwal R., Mukhtar H. (1993). Protection against malignant conversion of chemically induced benign skin papillomas to squamous cell carcinomas in SENCAR mice by a polyphenolic fraction isolated from green tea. Cancer Res..

[7] Takuo O., Kazuko M., Hikoya H. (1984). Inhibitory effect of tannins on direct-acting mutagens. Chem. Pharm. Bull..

[8] Lin T.C., Hsu F.L., Cheng J.T. (1993). Antihypertensive Activity of Corilagin and Chebulinic Acid, Tannins from Lumnitzera, racemose. J. Nat. Prod..

[9] Sasaki Y.F., Imanishi H., Ohta T., Watanabe M., Matsumoto K., Shirasu Y. (1988). Suppressing effect of tannic acid on UV and chemically induced chromosome aberrations in cultured mammalian cells. Agric. Biol. Chem..

[10] Iwawoto M., Uchino K., Toukairin T., Kawaguchi K., Tatebayashi T., Ogawara H., Tonosaki Y. (1991). The Growth Inhibition of Streptococcus mutans by 5′-Nucleotidase Inhibitors from Areca catechu L.. Chem. Pharm. Bull..

[11] Han W. (2007). Cuttage Technology Research of Rosa Hybrida and Rosa Banksiae. Anhui Agric. Sci. Bull..

[12] Wang M., Zhang C., Li M., Gao X. (2019). The complete chloroplast genome sequence of Rosa banksiae var. normalis (Rosaceae). Mitochondr. DNA Part B.

[13] Yu A., Wang X., Yang X. (2007). Chemical composition of the essential oils of flowers of Rosa banksiae from China. Chem. Nat. Compd..

[14] Nowak R., Tuzimski T. (2005). A solid-phase extraction-thin-layer chromatographic-fiber optical scanning densitometric method for determination of flavonol aglycones in extracts of rose leaves. J. Planar Chromatogr..

[15] Lee J.E., Kim G.S., Park S., Kim Y.H., Kim M.B., Lee W.S., Jeong S.W., Lee S.J., Jin J.S., Shin S.C. (2014). Determination of chokeberry (Aronia melanocarpa) polyphenol components using liquid chromatography-tandem mass spectrometry: Overall contribution to antioxidant activity. Food Chem..

[16] Konwarh R., Pramanik S., Kalita D., Mahanta C.L., Karak N. (2012). Ultrasonication—A complementary ‘green chemistry’ tool to biocatalysis: A laboratory-scale study of lycopene extraction. Ultrason. Sonochem..

[17] Higdon J.V., Frei B. (2003). Tea Catechins and Polyphenols: Health Effects, Metabolism, and Antioxidant Functions. Crit. Rev. Food Technol..

[18] Süzgeç-Selçuk S., Birteksöz A.S. (2011). Flavonoids of Helichrysum chasmolycicum and its antioxidant and antimicrobial activities. S. Afr. J. Bot..

[19] Rothwell J.A., Knaze V., Zamora-Ros R. (2017). Polyphenols: Dietary assessment and role in the prevention of cancers. Curr. Opin. Clin. Nutr..

[20] Goodarzi S., Tabatabaei M.J., Jafari R.M., Shemirani F., Tofighi Z. (2020). Cuminum cyminum fruits as source of luteolin-7- O -glucoside, potent cytotoxic flavonoid against breast cancer cell lines. Nat. Prod. Res..

